# Protein Kinase C Isozyme Immaturity/Deficiency in Cord Blood Monocytes and Neutrophils

**DOI:** 10.3390/ijms252111665

**Published:** 2024-10-30

**Authors:** Khalida Perveen, Antonio Ferrante

**Affiliations:** 1 Department of Immunopathology, SA Pathology at the Women’s and Children’s Hospital, North Adelaide, SA 5006, Australia; khalida.perveen@adelaide.edu.au; 2 Robinson Research Institute, Adelaide Medical School, University of Adelaide, Adelaide, SA 5005, Australia; 3 School of Biological Sciences, University of Adelaide, Adelaide, SA 5005, Australia

**Keywords:** cord blood T cells, monocytes, neutrophils, cytokines, allergy, childhood infection, PKC, immunodeficiency of immaturity

## Abstract

Reduced/deficient expression of Protein Kinase C (PKC)ζ in Cord blood (CB) T cells is associated with allergy development in children and a propensity to maintain an immature T-helper (Th)2 cytokine profile. In addition, other PKC isozymes are also low in CBTCs. Since previous studies have reported that cord blood/neonatal monocyte and neutrophil functions are significantly lower than cells from adults, it was of interest to see if the CBTC PKC levels were reflected in CB monocytes and neutrophils. Compared to adult blood, CB expresses low levels of PKCα, β2, ε, θ, μ, ζ and λ/ι in monocytes and PKCα, β2, η, θ, μ, ζ and λ/ι in neutrophils. The T-cell PKCζ levels were positively correlated with levels in CB monocytes but not in neutrophils. However, neither the monocytes nor the neutrophil PKCζ were associated with T-cell development towards a Th1 or Th2 cytokine propensity, based on the production of interferon-gamma and interleukin-4 in response to phytohemagglutinin and phorbol myristate acetate. The results demonstrate that some newborn babies display a deficiency in PKC isozymes in monocytes and neutrophils, as reported for T cells. However, unlike T cells, the PKCζ levels of the phagocytes did not correlate with regulation of development towards a Th1 or Th2 cytokine phenotype.

## 1. Introduction

Blood phagocytes, monocytes and neutrophils play important roles in immunity against infection. These use both the adaptive and innate elements of the immune system to phagocytose and destroy invading bacteria. They also possess the capability of migrating to infection sites in tissues where they deploy these functions. It has been established that these antimicrobial functions of monocytes and neutrophils are immature [1] and deficient in the neonate. Neonatal neutrophils have diminished functional responses, e.g., reduced migration towards chemoattractant, diminished rolling adhesion, fewer cells attaching to activated endothelium and reduced migration to subendothelial tissue [1,2], impaired neutrophil extracellular trap formation [3,4], reduced chemiluminescence and bactericidal [5] and fungicidal [6] capacity, reduced chemotaxis in monocytes and neutrophils [7], and reduced reactive oxygen species (ROS) production in primed neutrophils [8], and neonatal monocytes exhibit lower adherence and extravasation [9].

Our previous research has established that a proportion of babies are born with a low or ‘deficient’ level of several Protein Kinase C (PKC) isozymes in T cells [10,11,12,13]. Interestingly, low levels of CBTC PKCζ were associated with a high risk of developing allergic sensitization in childhood [14]. In addition, the low levels led to the development of T cells with a propensity to skew towards a T-helper (Th)2 cytokine pattern, supporting the development towards allergic responses [10,11]. When examining the published research, it is evident that as per T cells, only some neonates showed defective responses in chemotaxis and adherence functions [7,8]. Thus, we examined whether the low levels of PKC isozymes, seen in CBTC, were also experienced by CB monocytes and neutrophils.

## 2. Results

### 2.1. PKC Isozyme Expression in CB Monocytes and Neutrophils

**Figure 1:**
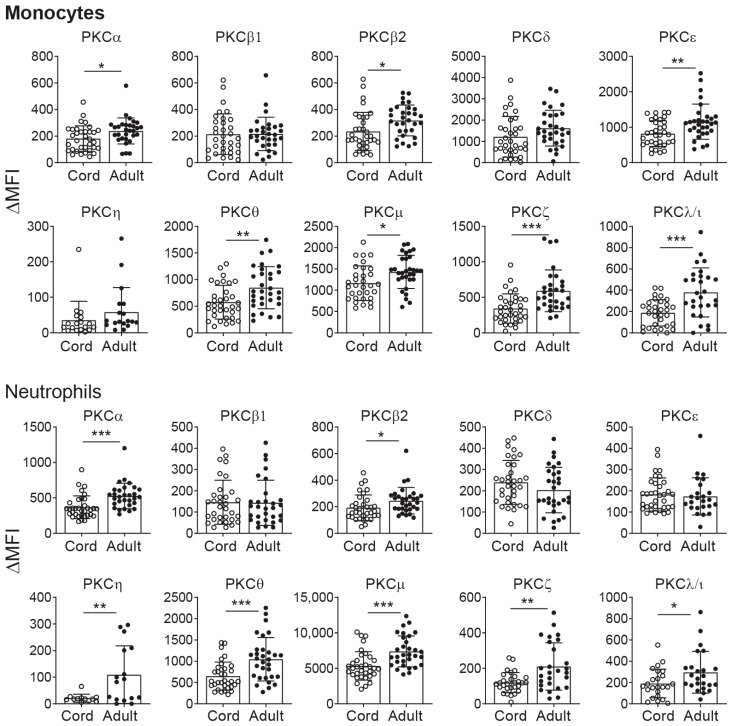
CB PKC isozymes expression in monocytes and neutrophils. Whole blood from the cord (*n* = 34) and adult donor blood (*n* = 31) was stained with anti-CD45 and anti-PKC isozymes antibodies, and gating of the subpopulations was based on their light scatter patterns for monocytes and neutrophils. PKC levels were quantified as change in median fluorescent intensity (ΔMFI) by flow cytometry, which were obtained after subtracting the isotype control MFI value from respective PKC isozymes MFI values. Data are presented as mean ± SD. * *p* < 0.05, ** *p* < 0.01, *** *p* < 0.001 (Student’s *t*-test).

The PKC family is composed of classical (α, β1, β2, γ), novel (ε, δ, θ, μ, η) and atypical (ζ, λ/ι) PKC isozymes. Previously, only T-cell PKC isozymes were reported to be deficient in CB leukocytes [10,14]. Here, we asked the question as to whether the levels of PKC isozymes are also deficient in CB monocytes and neutrophils. The levels of PKC isozymes were assessed using cord blood and adult blood by our previously established flow cytometry assays [10,11,12,13,15]. The data show that when compared to adult blood, CB monocytes are significantly lower or deficient in several of the PKC isozymes (Figure 1). CB monocytes expressed reduced levels of PKCα, β2, ε, θ, μ, ζ and λ/ι, while levels of PKCα, β2, ε, η, θ, μ, ζ and λ/ι were deficient in CB neutrophils (Table 1).

When a comparison was made between leukocyte subsets in CB, the levels of PKCβ2, μ, ζ and λ/ι were low in all cell types. Interestingly, expression of PKCα was reduced in CB monocytes and neutrophils but not in T cells or the T-cell subsets. But the opposite occurred for PKCδ, which was reduced in T cells but not in monocytes and neutrophils (Figure 1, Table 1). The levels of PKCη were low only in neutrophils (Figure 1, Table 1). Further assessment of low expression was made based on a cut-off below the 5th percentiles of the adult monocyte and neutrophil PKC isozyme levels. The findings are summarized in Table 2. Based on this criterion, it was evident that greater than 20% of the babies were born with PKCα, β2, ζ and λ/ι levels below the 5th percentile in monocytes. Almost half of the babies born had a deficient PKCβ2. For neutrophils, greater than 20% were born with PKCα, β2, η and μ lower than the 5th percentile.

We also compared the isozyme levels in male and female cord blood samples and found no significant difference between them apart from PKCβ2 in monocytes and PKCλ/ι in neutrophils (Figures S1 and S2).

### 2.2. Correlation Assessment of PKC Isozyme Levels Between CB Leukocyte Subsets

**Figure 2:**
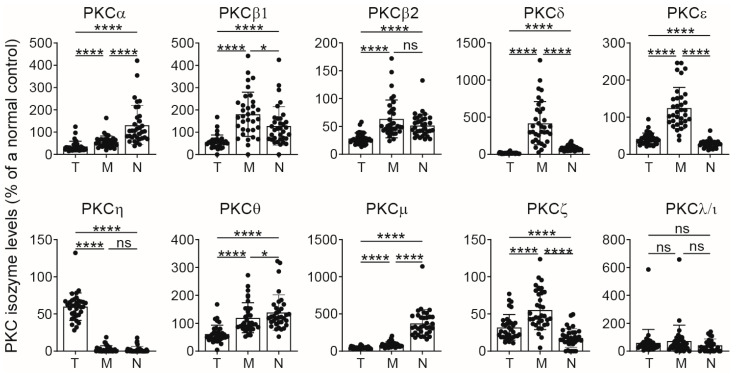
Comparison of PKC isozymes in CB T cells, monocytes and neutrophils using whole blood assays. The whole blood from cords was treated with anti-CD45 and anti-CD3 antibodies and co-stained for anti-PKC isozymes by intracellular staining by flow cytometry. Analysis of each PKC isozyme was performed by gating the subpopulation based on the expression of CD3 T cells (T, previously published data [11]) or their light scatter patterns for monocytes (M) and neutrophils (N). Bar graphs represent values for each individual and as mean ± SD (*n* = 34), expressed as change in ΔMFI, which were obtained after subtracting the isotype control MFI value from respective PKC isozymes MFI values and then expressed as the percentage of cryopreserved adult standard T-cells PKCs values. * *p* < 0.05, **** *p* < 0.0001. ns: not significant. One-way ANOVA with post hoc Tukey’s multiple comparisons test.

Having shown that PKC isozyme expression was low in T cells, monocytes and neutrophils in CB, it was important to see if this correlated among the three different leukocytes since accessory cells such as monocytes/macrophages as well as neutrophils can influence T-cell responses, and whether an individual neonate would face the challenge of both phagocytes being low/deficient. Reduced levels of T-cell PKC isozymes raised the question as to whether levels of PKC isozymes are comparable between different CB leukocyte subpopulations. Here, the levels in T cells, monocytes and neutrophils were examined in the same samples as in Figure 1 by using anti-CD45, anti-CD3 and anti-PKC isozyme antibodies. The data were normalized against the values of CD3^+^ T cells of a standard cryopreserved peripheral blood mononuclear cell (PBMC) sample [13], which was run concurrently with each assessment to ensure consistency between different runs, and all results were expressed as a percentage of this standard. The data presented in Figure 2 show that the CB monocytes expressed the highest levels of most of the PKC isozymes except for PKCα, θ and μ, which were highest in CB neutrophils. PKCη was mainly expressed in CBTC.

**Figure 3:**
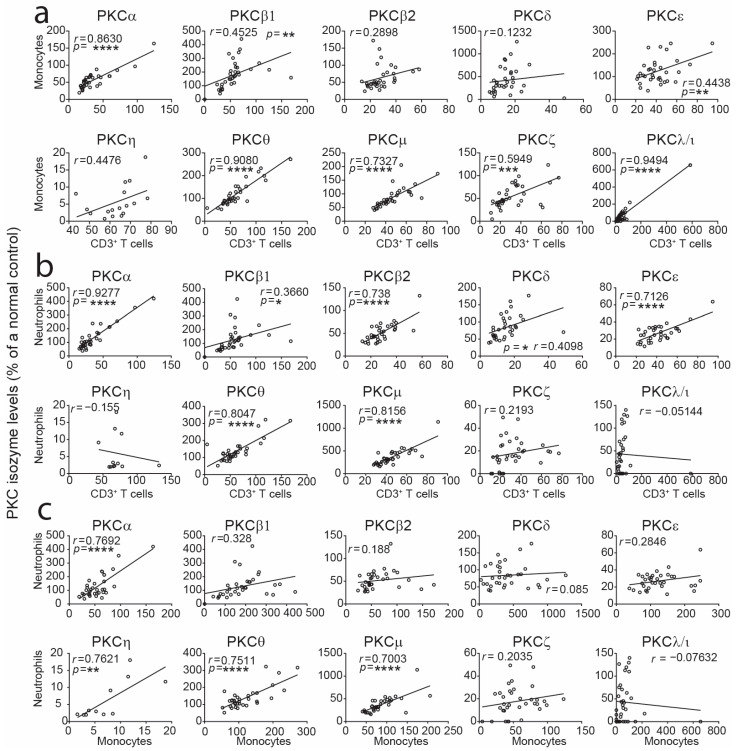
Correlation analyses of levels of PKC isozymes in monocytes or neutrophils with T cells. Data from Figure 2 were subjected to correlation analysis in T cells vs. monocytes (**a**), T cells vs. neutrophils (**b**) and monocytes vs. neutrophils (**c**). Correlations were performed using the two-tailed Pearson correlation coefficient. Data are presented as dots for the measurements of two variables from each of the samples and simple linear regression lines. * *p* < 0.05, ** *p* < 0.01, *** *p* < 0.001, **** *p* < 0.0001. CB *n* = 34.

We asked whether there was any correlation between levels of PKC isozymes in CBTC, monocytes and neutrophils. Comparing the levels between CBTC and monocytes, PKCα, β1, ε, θ, μ, ζ and ι/λ were positively correlated, and PKCα, β1, β2, δ, ε, θ and μ were significantly correlated between CB T cells and neutrophils (Figure 3). There was a positive correlation in PKCα, η, θ and μ between CB monocytes and neutrophils (Figure 3).

### 2.3. Correlation Assessment Between CB Monocyte PKCζ Expression and T-Cell Cytokines

**Figure 4:**
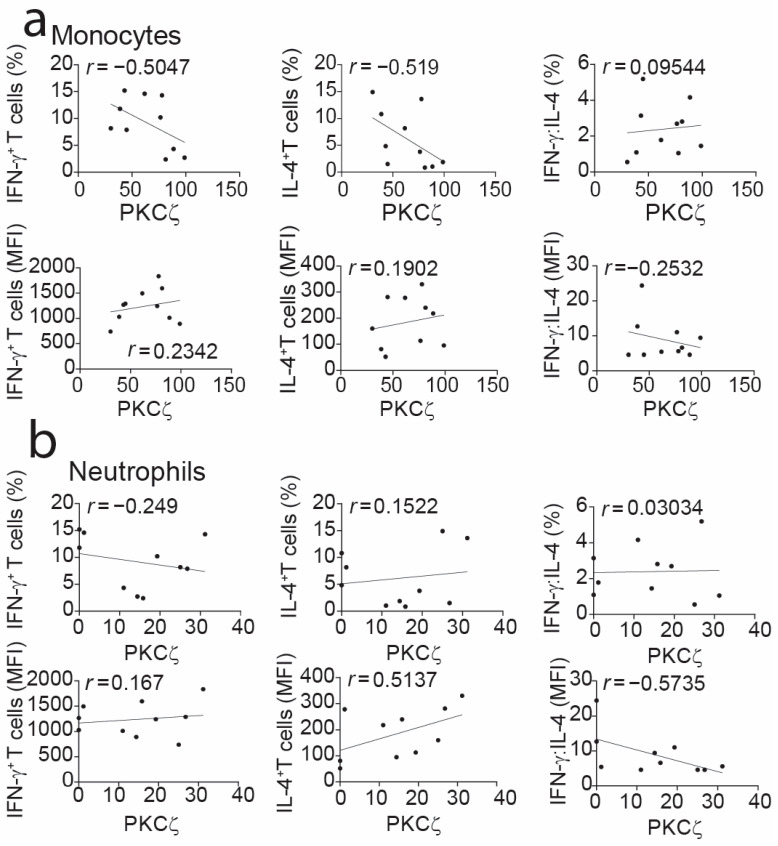
Correlation analyses of expression levels of PKCζ in cord blood leukocytes vs. cytokine production in matured CD3^+^ T cells. PKCζ data in monocytes (**a**) and neutrophils (**b**) were subjected to correlation analysis with cytokine production in in vitro matured CD3^+^ T cells. The results are expressed as PKCζ values as MFI in leukocytes vs. the percentage positive T cells ((**a**,**b**): top row panels) producing IFN-γ, IL-4 and a ratio of IFN-γ:IL-4 or MFI levels ((**a**,**b**): bottom row panels) in the matured CBTC. Correlations were performed using the two-tailed Pearson correlation coefficient. CB *n* = 10. Data are presented as dots for the measurements of two variables from each of the samples and simple linear regression lines.

Previous studies were conducted on purified or gating on CBTC, to study the relationship between expression of PKCζ levels in immature T cells and cytokines produced by the matured T cells [10,11]. In these experiments, to see if the matured T cells were skewed towards a Th1 or Th2 cytokine bias, maturation was induced by treatment with phytohemagglutinin (PHA) and interleukin-2 (IL-2). The matured T cells were stimulated with PHA and phorbol 12-myristate 13-acetate (PMA) and then examined for levels of cytokines by intracellular staining with monoclonal antibodies and application to flow cytometry [10,11,13,15]. Examining the association between cytokine patterns and PKCζ levels in CB monocytes revealed no strong relationship. This was true for interferon-gamma (IFN-γ), IL-4 or their ratios, assessed by both the percentage of positive cells and MFI amounts (Figure 4). While this was unexpected, as there was a correlation between the levels of this isozyme between T cells and monocytes, it is possible that the very much higher expression in monocytes may have masked the association. Also, the data showed no correlation between the PKCζ levels in neutrophils and the skewing of the Th responses (Figure 4). This emphasizes the importance of CBTC PKCζ per se being responsible for the Th skewing.

## 3. Discussion

Neonatal monocytes and neutrophils display deficient antimicrobial activity compared to cells from adult blood [1,7]. The concern is that such a compromised/immaturity of neonatal phagocytes makes the newborn more susceptible to infections, particularly if preterm [6]. While CB monocytes and neutrophils express similar or even higher levels of most signaling proteins involved in cell activation and inflammation, CB monocytes specifically show comparable expression levels of ERK2 and p38 proteins. They also exhibit equal or enhanced phosphorylation of ERK1/2 and p38-mitogen-activated protein kinase in response to bacterial lipopolysaccharide or peptidoglycan stimulation [16,17]. Similarly, CB neutrophils express comparable levels of protein tyrosine kinase p53/56^lyn^ and increased granule and membrane levels of ERK2 and p38 proteins [18], relative to adult controls. The functional differences in these signaling molecules are believed to stem from variations in their localization and activity within the cells.

Our data demonstrate for the first time that both neonatal monocytes and neutrophils express all of the different isozymes of PKC but that both cell types from cord blood have significantly low PKC isozyme expression. However, the type and levels of deficiency in different PKC isozymes differed in each cell type examined. Furthermore, these deficiencies in PKC isozymes do not correlate in all cell types examined. While the consequences of these deficiencies remain to be studied, the role of these isozymes in phagocyte function has been reported. Many of the PKC isozymes have been reported to play a role in neutrophils’ bactericidal functions, e.g., the phospholipase C/PKC/PKD signaling axis is involved in G Protein-Coupled receptor-mediated chemotaxis of neutrophils [19]; PKCα, β and δ promote ROS production; and PKCβII and PKCζ are involved in cytoskeleton remodeling [20]. Classical PKCs, especially PKCβ, have a prominent role in NET formation [21]. Atypical PKCζ mediates *N*-formyl-methionyl-leucyl-phenylalanine-induced phosphorylation of p47(phox) and nicotinamide adenine dinucleotide phosphate oxidase activation to generate superoxide anion [22], activation of peptidyl arginine deiminase 4 (PAD4) and the execution of NETosis [23], and adhesion and chemotaxis [24]. Data on gender differences in CB PKC isozymes revealed significantly higher levels of only PKCβ in the monocyte population and PKCλ/ι in neutrophils from female CB samples compared to males, and this needs to be taken into consideration when emphasizing the deficiency in neonates.

Thus, it will be of interest if the low expression of these PKC isozymes can explain the reduced ability to phagocytose and produce ROS, crucial for killing bacteria, diminished chemotaxis/migration to sites of infection, dampened innate immunity because of less effective signaling via the Toll-like receptors [25], as reported in newborn neutrophils that have a diminished priming response to lipopolysaccharide (Toll-like receptors-4 ligand) due to altered protein tyrosine kinase activities; reduced nuclear binding activity of Nuclear factor kappa-light-chain-enhancer of activated B cells induced by *N*-formyl-methionyl-leucyl-phenylalanine [26]; and in the case of monocytes, less production of inflammatory cytokines, IFN-γ, IL-1β, tumor necrosis factor and IL-12, required for promoting the adaptive immune response [25,27].

Intriguingly, all three leukocyte types display low levels of PKC isozymes in the newborn (cord blood). A closer analysis of the data demonstrated that as per T cells, a proportion of the babies show a deficiency based on being <5th percentile of normal (adult values). In the case of CBTC PKCζ, the low expression was linked to influencing the maturation of T cells towards a Th2 cytokine profile and allergy risk [10,11,14]. Indeed, the levels of PKCζ in CBTC correlated with T cells maturing towards either a Th1 or Th2 cytokine production propensity [10,11,14], including the importance of the PKCζ signaling pathway in the regulation of this T-cell development [15]. Our present data support a view that CBTC PKCζ levels per se control their development. Accordingly, we found no correlation between PKCζ CB monocyte levels and skewing of the Th cells, similarly for CB neutrophils.

Expression of several of the PKC isozymes was low: PKCα, β2, θ, μ, ζ and λ/ι, in both monocytes and neutrophils. PKCε and PKCη were significantly deficient only in CB monocytes and neutrophils, respectively. It is evident that a large number of the CB monocytes and neutrophil PKC levels are low, i.e., <5th percentile. Interestingly, examination of the published data on neonatal neutrophil and monocyte functional responses also shows that a proportion of CB cells also has low responses of antimicrobial activity [7,28]. Thus, the present study and our previously reported work on CBTC emphasize the importance of considering individual newborns for PKC deficiency or immaturity. Consequently, the published data on functional responses of monocytes and neutrophils require this per individual emphasis.

## 4. Materials and Methods

### 4.1. Reagents

In the present study, the following reagents were used, with the companies that they were purchased from stipulated in parentheses: X-VIVO 15 media (04418Q, Lonza, Basel, Switzerland); RPMI 1640 tissue culture medium, DMSO, PHA, PMA and human AB serum (Sigma Aldrich, St. Louis, MO, USA); fetal calf serum and L-glutamine (AFC Biosciences, Lenexa, KS, USA); rhIL-2 (PeproTech, Rocky Hill, NJ, USA).

### 4.2. Ethics Statement

The procurement of human blood and all experimental procedures were approved by the Human Research Ethics Committee of the Women’s and Children’s Health Network (WCHN), Adelaide, South Australia, in accordance with The National Statement on Ethical Conduct in Human Research (2007, updated 2018) (National Health and Medical Research Council Act 1992). Venous blood was collected from healthy adult volunteers with their informed consent and umbilical CB from healthy neonates with informed consent from pregnant women undergoing elective caesarean section.

### 4.3. Collection of Blood Samples and Isolation of Mononuclear Cells

Cord blood samples (*n* = 34) were collected following elective caesarean sections (Gestation age 37–39 weeks, 17 females and 18 males) with no complications at birth. These samples were analyzed for the PKC isozyme levels within 2 h of collection. Additionally, cord blood mononuclear cells (CBMCs) or PBMC were prepared by centrifuging blood samples over Ficoll^®^ Paque Plus and cryopreserved in freezing media (90% heat-inactivated fetal calf serum and 10% DMSO), as previously described [13] for later functional analysis.

### 4.4. Maturation of Cord Blood T Cells in Culture

CBTC maturation was conducted as previously described [13]. Briefly, CBMC at 1 × 10^6^/mL in complete media (RPMI-1640 medium supplemented with 10% fetal calf serum, 2 mmol/L L-glutamine, 100 μg/mL streptomycin and 100 U/mL penicillin) was seeded in a 12-well plate. The cells were matured by adding PHA (2 μg/mL) on day 1 and IL-2 (10 ng/mL) on day 3, then every 2nd day, when the media was replaced with fresh media. On day 7, the matured T cells were stimulated with PHA-PMA and examined for cytokine production.

### 4.5. Measurement of PKC Isozyme Expression by Flow Cytometry

The expression of PKC isozymes was assessed as described previously [13]. Briefly, whole blood was surface-stained with anti-CD3 APC-H7 and anti-CD8 PE-Cy7, both from BD Biosciences (Franklin Lakes, NJ, USA), for 20 min, followed by fixation with BD Cytofix/Cytoperm (BD, 555028) and permeabilization with NET-Gel. The cells were treated with mouse/rabbit IgG (1 µg) Fc blocking reagent, followed by addition of fluorochrome-labeled isotype controls or anti-PKC isozyme antibodies (Table 3) for 30 min at room temperature (RT) in the dark. The cells were analyzed on a FACS Canto II (BD Biosciences, Franklin Lakes, NJ, USA); data were assessed using FlowJo v10.1 (Ashland, OR, USA) on cell populations with the following gating strategy: doublets excluded using a forward scatter-area (FSC-A) versus—height (FSC-H) plot, then lymphocyte, monocyte and neutrophil populations were ascertain based on their side scatter-area (SSC-A) versus FSC-A. PKC isozymes were gated positive based on fluorescence minus one and isotype controls. The data are presented as median fluorescent intensities (MFIs), which were obtained after subtracting the isotype control MFI value from respective PKC isozymes MFI values.

### 4.6. Measurement of Intracellular Cytokines

The assays were conducted essentially as previously described using the BD Cytofix/Cytoperm Plus Permeabilization Kit with GolgiPlug [10]. Briefly, 1 × 10^6^/mL T cells was stimulated with 2 µg/mL of PHA and 50 nM of PMA in culture media RPMI-1640/2.5% AB serum containing Brefeldin A (GolgiPlug) for 16–20 h at 37◦C/5% CO_2_. At the end of incubation, cells were washed and surface-stained for 20 min, followed by fixation (20 min), and permeabilized (10 min) at RT in the dark. Cells were stained with fluorochrome-conjugated antibodies to detect intracellular cytokine (see Table 4) for 30 min at RT in the dark. The samples were washed twice and analyzed on a FACS Canto II.

### 4.7. Statistical Analysis

Statistical comparisons were performed using one-way ANOVA with a post hoc Tukey’s multiple comparisons test, or Student’s *t*-testing. Correlations were performed using the two-tailed Pearson correlation coefficient. All statistical analyses were performed using GraphPad Prism v10 (GraphPad Software, La Jolla, CA, USA). A *p*-value of <0.05 was considered statistically significant for all analyses.

## 5. Conclusions

The expression of several PKC isozymes was found to be low or deficient in CB monocytes and neutrophils compared to cells from adults. This highlights a new finding with respect to neonatal immunity. It may explain the previously reported reduced functional responses of neonatal neutrophils and monocytes. This work extends our previous findings that neonatal T cells show significantly reduced expression of PKC isozymes. It may also be tentatively concluded that the reports that PKCζ levels in neonatal T cells correlate with the maturation towards Th1 cytokine production is intrinsic to T cells and not influenced by the levels in monocytes or neutrophils.

## 6. Study Limitation

While the findings report that the levels of several PKC isozymes are critically low in cord blood neutrophils and monocytes, it remains to be demonstrated directly if the deficiencies are linked to the compromised antimicrobial functional responses of these cells, including the potential to differentiate towards an M1 or M2 macrophage functional phenotype. This study also assumes that the phosphorylation/activation of the PKC isozymes is not affected by the immaturity and may need further investigations.

## Figures and Tables

**Table 1:** Comparison of PKC isozymes in cord vs. adult blood leukocytes.

Cell Type	PKCα	PKCβI	PKCβII	PKCδ	PKCε	PKCη	PKCθ	PKCμ	PKCζ	PKCλ/ι
CD4	-	-	*** ↓	**** ↓	-	-	-	* ↓	** ↓	*** ↓
CD8	-	-	**** ↓	**** ↓	** ↓	-	** ↓	* ↓	**** ↓	**** ↓
CD3	-	-	**** ↓	**** ↓	* ↓	-	* ↓	* ↓	**** ↓	**** ↓
Monocytes	* ↓	-	* ↓	-	** ↓	-	** ↓	* ↓	*** ↓	*** ↓
Neutrophils	*** ↓	-	* ↓	-	-	** ↓	*** ↓	*** ↓	** ↓	* ↓

Whole blood from the cord (*n* = 34) and adult donor blood (*n* = 31) was stained with anti-CD45 and anti-PKC isozymes antibodies. Gating of the monocytes and neutrophils subpopulations was based on their light scatter patterns. Data on CD3, CD4 and CD8 cells are from the previously published paper [11]. * *p* < 0.05, ** *p* < 0.01, *** *p* < 0.001, **** *p* < 0.0001 (Student’s *t*-test). Show significantly decreased (↓) from adult leukocyte subpopulation levels.

**Table 2:** Percentage of CB samples expressing PKCζ levels below 5th percentile.

	Monocytes			Neutrophils		
PKC	5th % Cut-Off Adults (MFI) *	No. CB (<5th %) ^†^	% of CB ^‡^	5th % Cut-Off Adults (MFI) *	No. CB (<5th %) ^†^	% of CB ^‡^
PKCα	38	8	24	77	11	33
PKCβ1	44	1	3	32	0	0
PKCβ2	49	15	45	37	8	24
PKCδ	152	5	15	13	0	0
PKCε	53	2	6	10	0	0
PKCη	2	3	9	4	8	24
PKCθ	66	3	9	63	1	3
PKCμ	50	4	12	249	9	27
PKCζ	39	10	30	7	1	3
PKCλ/ι	31	7	21	25	5	15

* The data show the adult blood 5th-percentile cut-off values for each PKC isozyme as ΔMFI, which are expressed as the percentage of cryopreserved adult control T-cells PKCs values (standards). ^†^ shows the number of CB samples below the 5th-percentile cut-off of adult values, and ^‡^ shows the percentage of CB samples out of a total *n* = 34 CB samples tested showing levels below the 5th-percentile cut-off of adult values.

**Table 3:** Staining panel for the determination of PKC isozyme expression.

Antibody	Fluorochrome	Clone	Catalogue
Anti-PKCα	AF647	H-7 ^1^	sc-8393
Anti-PKCβ1	AF647	EPR18512 ^2^	ab223452
Anti-PKCβII	AF647	F-7 ^1^	sc-13149
Anti-PKCθ	PE	E-7 ^1^	sc-1680
Anti-PKCε	AF488	EPR1482(2) ^2^	ab217980
Anti-PKCδ	AF488	EPR17075 ^2^	ab206282
Anti-PKCη	AF488	EPR18513 ^2^	ab179524
Anti-PKCμ	AF647	EP1493Y ^2^	ab51246
Anti-PKCζ	PE	H-1 ^1^	sc-17781
Anti-PKCλ/ι	PE	H-12 ^1^	sc-17837
Rabbit mAb IgG Isotype Control	AF647	- ^4^	2985
Rabbit mAb IgG Isotype Control	AF488	- ^4^	2975
Mouse IgG1k Isotype control	AF647	MOPC-31C ^3^	566011
Mouse mAb IgG2ak	PE	X39 ^3^	340459

^1^ Santa Cruz Biotechnology (Dallas, TX, USA), ^2^ Abcam (Cambridge, UK), ^3^ BD Biosciences (Franklin Lakes, NJ, USA), ^4^ Cell Signaling Technologies (Danvers, MA, USA).

**Table 4:** Intracellular staining of IL-4 and IFN-γ cytokines in T cells.

Antibody	Fluorochrome	Clone	Catalogue
Anti-CD3	PE-CY5	HIT3a ^1^	555341
Anti-CD45	APC-H7	2D1 ^1^	641399
Anti-IFN-γ	FITC	4S.B3 ^1^	554551
Anti-IL-4	PE	8D4-8 ^2^	12-7049-42
Mouse-IgG1k	FITC	MOPC-21 ^1^	555748
Mouse-IgG1k	PE	MOPC-21 ^1^	556650

^1^ BD Biosciences (Franklin Lakes, NJ, USA), ^2^ eBiosciences (San Diego, CA, USA).

## Data Availability

The datasets generated and analyzed during the current study are available from the corresponding author upon reasonable request.

## Supplementary Materials

The following supporting information can be downloaded at: https://www.mdpi.com/article/10.3390/ijms252111665/s1.
