# Supplementary material for: Protein Kinase C Isozyme Immaturity/Deficiency in Cord Blood Monocytes and Neutrophils
Source: Int J Mol Sci. 2024 Oct 30;25(21):11665. doi: 10.3390/ijms252111665 (PMC11546585; doi:10.3390/ijms252111665)
Supplement: Supplementary file 1 [file ijms-25-11665-s001.zip › ijms-3255885-supplementary.pdf]

Article

Protein Kinase C Isozyme Immaturity/Deficiency in Cord Blood Monocytes and Neutrophils

Khalida Perveen [1,2] and Antonio Ferrante [1,2,3,*]

[1] Department of Immunopathology, SA Pathology at the Women's and Children's Hospital, North Adelaide, SA 5006, Australia; khalida.perveen@adelaide.edu.au
[2] Robinson Research Institute, Adelaide Medical School, University of Adelaide, Adelaide, SA 5005, Australia
[3] School of Biological Sciences, University of Adelaide, Adelaide, SA 5005, Australia
* Correspondence: antonio.ferrante@adelaide.edu.au; Tel.: +61-8-81-617-216

Supplementary data
This file includes:
Figures S1 and S2

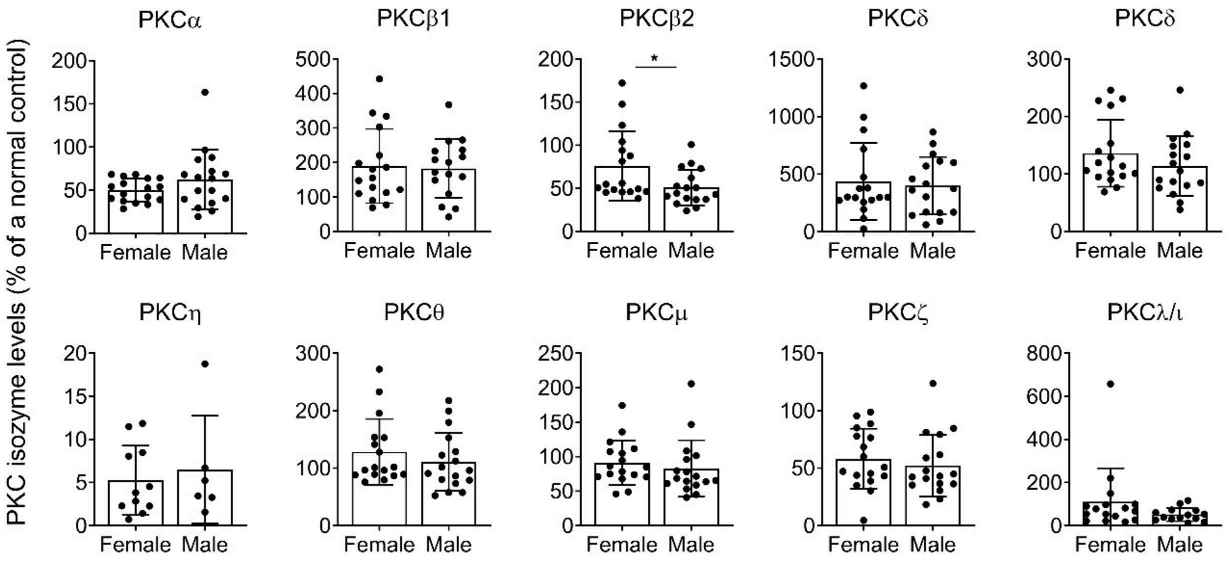

Figure S1. Comparison of PKC isozyme levels in CB monocytes between females and males. Data from Figure 2 were used to analyse PKC isozyme levels in females vs males. Bar graphs represent values for each individual and as mean ± SD (n=17 each group), expressed as change in MFI which were obtained after subtracting the isotype control MFI value from respective PKC isozymes MFI values and then expressed as percentage of cryopreserved adult standard T cells PKCs values. * $p < 0.05$. (Student's t-test).

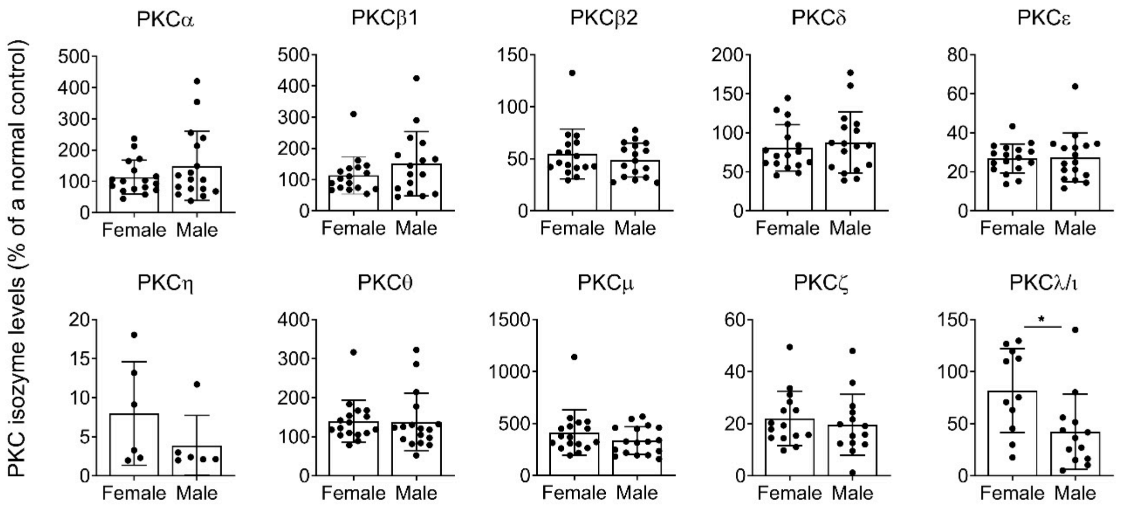

Figure S2. Comparison of PKC isozyme levels in CB neutrophils between females and males. Data from Figure 2 were used to analyse PKC isozyme levels in females vs males. Bar graphs represent values for each individual and as mean ± SD (n=17 each group), expressed as change in MFI which were obtained after subtracting the isotype control MFI value from respective PKC isozymes MFI values and then expressed as percentage of cryopreserved adult standard T cells PKCs values. * $p < 0.05$. (Student's t-test).
